# Unique microglia recovery population revealed by single-cell RNAseq following neurodegeneration

**DOI:** 10.1186/s40478-018-0584-3

**Published:** 2018-09-05

**Authors:** Tuan Leng Tay, Sagar, Jana Dautzenberg, Dominic Grün, Marco Prinz

**Affiliations:** 1 Institute of Neuropathology, Faculty of Medicine, University of Freiburg, Freiburg, Germany; 2 Cluster of Excellence BrainLinks-BrainTools, University of Freiburg, Freiburg, Germany; 3 Institute of Biology I, Faculty of Biology, University of Freiburg, Freiburg, Germany; 4 Max-Planck-Institute of Immunobiology and Epigenetics, Freiburg, Germany; 5 BIOSS Centre for Biological Signaling Studies, University of Freiburg, Freiburg, Germany

**Keywords:** Microglia, Recovery, Neurodegeneration, Single-cell RNA analysis

## Abstract

Microglia are brain immune cells that constantly survey their environment to maintain homeostasis. Enhanced microglial reactivity and proliferation are typical hallmarks of neurodegenerative diseases. Whether specific disease-linked microglial subsets exist during the entire course of neurodegeneration, including the recovery phase, is currently unclear. Taking a single-cell RNA-sequencing approach in a susceptibility gene-free model of nerve injury, we identified a microglial subpopulation that upon acute neurodegeneration shares a conserved gene regulatory profile compared to previously reported chronic and destructive neurodegeneration transgenic mouse models. Our data also revealed rapid shifts in gene regulation that defined microglial subsets at peak and resolution of neurodegeneration. Finally, our discovery of a unique transient microglial subpopulation at the onset of recovery may provide novel targets for modulating microglia-mediated restoration of brain health.

**Electronic supplementary material:**

The online version of this article (10.1186/s40478-018-0584-3) contains supplementary material, which is available to authorized users.

## Introduction

Microglia are tissue-resident macrophages of the central nervous system (CNS) that act as the first line of defense upon disruption of CNS homeostasis. In contrast to the lattice-like organization of sparsely (< 0.5%) renewing microglial cells in the adult brain [3, 26, 27, 35, 43], heightened microglial reactivity and microgliosis are hallmarks of all neurodegenerative diseases regardless of severity, as exemplified in local neuronal damage and widespread neurodegeneration [10, 13, 32, 37, 43].

While adult microglia originate solely from the primitive yolk sac erythromyeloid progenitors without contribution from the peripheral hematopoietic stem cells [1, 11, 12, 22, 33], gene expression and single-cell transcriptomic studies [14, 29] suggest that total CNS parenchymal microglia are not functionally homogeneous. The relative contributions to neuroprotection and neurodegeneration by microglia in neurodegenerative diseases such as Alzheimer’s disease (AD), amyotrophic lateral sclerosis and multiple sclerosis remain contentious [38, 39]. Notably, we recently demonstrated that immediate activation and proliferation of microglial cells within one to two weeks of neuronal injury was not detrimental to the CNS but appeared vital to the timely recovery of tissue homeostasis and neural functions [43]. Bulk RNA-sequencing (RNAseq) analyses of microglial cells of the facial nucleus (FN) from the unilateral facial nerve axotomy (FNX) model of acute neurodegeneration showed lesion-dependent gene regulation, while compensatory alterations observed in the contralateral FN were attributed to other CNS cell types [43]. Recent reports based on single-cell analysis of microglial transcriptomes attributed specific cellular states to neurodegenerative diseases recapitulated in AD-like mouse models with chronic or severe CNS damage [21, 30]. Although these important studies highlighted the appearance of novel disease-associated microglial subtypes, they did not address the existence of distinct microglial populations during recovery due to the chronic and destructive characteristics of the transgenic mouse models used.

To define disease-associated populations of microglia more precisely, we took a single-cell RNAseq (scRNAseq) approach in the FNX model, which is not driven by any susceptibility gene. Indeed, a subset of disease-linked microglia from the ipsilateral FN was distinct from a homogenous cloud. Comparative analysis of single-cell transcriptomes across these three models of neurodegeneration furthermore established a strong conservation of the microglial gene regulatory profile ascribed to disease. Of high significance, we found temporal regulation of lesion-associated changes in our FNX model that distinguished microglia at peak and resolution of disease. In particular, we verified the emergence of a transient microglial cluster characterized by the upregulation of *Apoe* and *Ccl5* at the onset of recovery in situ. Collectively, our findings highlight a potential new interpretation of disease-associated gene regulation that may be critical to the restoration of CNS homeostasis mediated by microglial cells.

## Materials and methods

### Mice and treatments

*CX*_*3*_*CR1*^*GFP/+*^ [20] mice were bred in specific-pathogen-free facility and given chow and water ad libitum. Unilateral facial nerve axotomy (FNX) at the stylomastoid foramen was performed in 8 weeks old female *CX*_*3*_*CR1*^*GFP/+*^mice described previously [43]. Only female mice were used to allow comparisons of the scRNAseq data in this study with the bulk RNAseq analyses performed before [43]. Mice were bred concurrently, received same-day operation and randomly assigned to each experimental group for sacrifice at the required time point. Animal experiments were approved by the Regional Council of Freiburg, Germany. Experimenters were blinded to all groups during data acquisition and analysis.

### FACS

Mice were transcardially perfused with 20 ml ice-cold PBS. Pontine blocks were immediately cut in a coronal rodent brain matrix for acute isolation of single facial nuclei under the stereomicroscope. Brain tissue was gently mashed and resuspended in 20 ml ice-cold extraction buffer containing 1× HBSS, 1% fetal calf serum (FCS) and 1 mM EDTA, followed by the extraction of microglial cells in 5 ml 37% isotonic Percoll. Cells were labeled with antibodies CD45-BV421 (103,133, BioLegend), CD11b-BV605 (101,237, BioLegend) and MHC Class II-PE-Cy7 (107,630, BioLegend) in FACS buffer (1× PBS, 1% FCS). Single GFP^+^ CD45^lo^ CD11b^+^ microglial cells were sorted into 384-well plates containing 240 nL of primer mix and 1.2 μl of Vapor-Lock (QIAGEN) PCR encapsulation barrier at the Influx™ cell sorter (Becton Dickinson) for subsequent RNA sequencing procedures.

### Single-cell RNA amplification and library preparation

We used an automated and miniaturized version of the CEL-Seq2 protocol [18]. Sixteen libraries (1536 single cells) were sequenced on two lanes (pair-end multiplexing run, 100 bp read length) of an *Illumina HiSeq* 2500 sequencing system generating 243,638,747 sequence fragments.

### Quantification of transcript abundance

For the FNX experiment, paired end reads were aligned to the transcriptome using bwa (version 0.6.2-r126) with default parameters [28]. The transcriptome contained all RefSeq gene models based on the mouse genome release mm10 downloaded from the UCSC genome browser comprising 31,201 isoforms derived from 23,538 gene loci [31]. All isoforms of the same gene were merged to a single gene locus. The 50 bp right mate of each read pair was mapped to the ensemble of all gene loci and to the set of 92 ERCC spike-ins in sense direction [4]. Reads that mapped to multiple loci were discarded. The 50 bp left read contains the barcode information: the first six bases corresponded to the unique molecular identifier (UMI) followed by six bases representing the cell specific barcode. The remainder of the left read contains a polyT stretch. Only the right read was used for quantification. For each cell barcode, the number of UMIs per transcript was counted and aggregated across all transcripts derived from the same gene locus. Based on binomial statistics, the number of observed UMIs was converted into transcript counts [15].

### Single-cell RNA sequencing data analysis

Identification and visualization of different subpopulations as well as differential gene expression analysis was performed with the RaceID2 algorithm [16]. Out of 1536 cells sequenced in the FNX experiment, 944 cells passed the quality thresholds. The median, minimum and maximum number of genes identified per cell are 1560, 858 and 2658, respectively. Down-sampling to 1500 transcripts was used for data normalization. Clustering was performed using k-medoids clustering without outlier identification. Ten clusters were identified based on the saturation of the average within-cluster dispersion. To compare our disease-associated clusters with a recently described microglia type associated with neurodegenerative disease (DAM), we obtained the raw data from scRNAseq of all immune cells in wild type (WT) and Alzheimer’s disease (AD) transgenic mouse brains [21]. The AD mouse model expressed five human familial AD gene mutations (FAD). Results were obtained from a mix of male and female mice which showed no difference due to sex. Raw count files (henceforth referred to as the “FAD data set”) were downloaded from Gene Expression Omnibus (GEO): GSE98969 [21] and analyzed using the RaceID2 algorithm [16]. To exclude non-microglial cells from the FAD data set, only cells with UMI counts for *Cst3* (UMI > 10) and *Hexb* (UMI > 5) (as defined in [21]) prior to normalization were retained for further analysis. Perivascular macrophages and monocytes (*Cd74*, UMIs ≥5), granulocytes (*S100a9*, UMIs ≥50) and mature B-cells (*Cd79b*, UMIs ≥3) were removed from the dataset. Downsampling to 700 UMIs was performed for data normalization.

The t-distributed stochastic neighbor embedding (t-SNE) algorithm was used for dimensional reduction and cell cluster visualization [44]. Using the *phyper* function provided by the R software to perform a hypergeometric test, an enrichment score [−log_10_(*p*-value+ 10^− 3^)] was calculated for the FNX data to identify the enrichment of cells belonging to a group in a given cluster. Differentially expressed genes between the tail clusters (clusters 4, 8 and 9 in the FNX data) and cloud clusters were identified similar to a published method [2]. First, negative binomial distributions reflecting the gene expression variability within each subgroup were inferred based on the background model for the expected transcript count variability computed by RaceID2 [16]. Using these distributions, a *P* value for the observed difference in transcript counts between the two subgroups was calculated and multiple testing corrected by the Benjamini-Hochberg method.

The accession code for the FNX data set is GEO:GSE90975, https://www.ncbi.nlm.nih.gov/geo/query/acc.cgi?acc=GSE90975.

### Gene set enrichment analysis

Gene IDs of the differentially expressed genes between the tail (clusters 4, 8 and 9) and cloud clusters in the FNX data were converted to Entrez IDs using the clusterProfiler package [46]. Gene set enrichment analysis was performed using the ReactomePA package [45]. The fold-change for each gene between the cloud and tail clusters was calculated using the diffexpnb function of the RaceID2 algorithm and given as an argument to the gsePathway function to calculate enriched gene sets in the tail clusters.

### Comparative single-cell transcriptomic analysis

In addition to the comparison of our FNX data set with the DAM signature from the FAD scRNAseq study [21], we included the neurodegeneration response genes identified in another recent scRNAseq report based on the transgenic mouse model for severe neurodegeneration known as CK-p25 [30]. Male CK-p25 mice were analyzed. Withdrawal of doxycycline from the diet induces the CamKII promoter driven expression of p25, the calpain cleavage product of Cdk5 activator p35, and leads to apoptotic neuronal cell death. While the CK-p25 inducible mouse model is not based on genetic mutations associated with familial AD, the authors claimed that it recapitulates several aspects of AD pathology and the transcriptional profile of FAD mice [30]. Data on neurodegeneration-associated differentially regulated genes identified in the respective FAD and CK-p25 studies were obtained from the Supplementary Table S3 (fold changes and *P* values; [21]) and Supplementary Table S4 (fold changes and Z scores, from which *P* values were calculated for the corresponding early and late response genes in Clusters 3 and 6; [30]). A total of 5820 genes were obtained after we overlapped the relevant genes in both studies with our FNX gene set for comparative assessment. Based on the Benjamini-Hochberg procedure, only genes with false discovery rate < 0.05 were considered for subsequent analysis.

### Histology

Mice were transcardially perfused with 20 ml PBS. Brains were fixed overnight in 4% paraformaldehyde in PBS at 4°C and processed for frozen sectioning as before [43]. A coronal rodent brain matrix (RBM-2000C, ASI Instruments) was used to obtain consistent blocks of pontine regions that included both facial nuclei. Cryosections (14-μm) were collected from the entire facial nuclei on coated glass slides and stored at − 20°C until use. Tissues were permeabilized in blocking solution (0.1% Triton-X 100, 5% bovine albumin, normal goat or normal donkey serum, and PBS) for 1 h at room temperature and incubated overnight at 4°C with primary antibodies: 1:200 rat anti-CD11b (ab8878, Abcam), 1:500 rabbit anti-IBA-1 (019–19,741, Wako), 1:200 goat anti-APOE (AB947, Merck), and 1:500 rabbit anti-CCL5 (RANTES) (710,001, ThermoFisher Scientific). Antigen retrieval was performed at 96 °C prior to APOE staining for 40 min in 10 mM citrate buffer at pH 9. Sections were incubated with corresponding secondary antibodies conjugated to 1:1000 Alexa Fluor 488 or Alexa Fluor 647 (Life Technologies) and 1:5000 nuclear counterstain 4′,6-diamidino-2-phenylindole (DAPI, Sigma) for 2 h at room temperature, and mounted in ProLong® Diamond Antifade Mountant (Life Technologies).

### Microscopy and image analysis

GFP^+^ microglia were imaged using a 20X / 0.75 NA objective lens on the Keyence BZ − 9000 inverted fluorescence microscope and quantified using the BZ-II Analyzer. Three brain sections per mouse were analyzed. Confocal images of immunohistological preparations were acquired with the SP8 STED-WS (Leica Microsystems) using a HCX PL HCL PL APO C 20X/0.75 NA glycerine objective lens and the LAS X software. DAPI and Alexa Fluors 488 and 647 were excited by the UV Diode Laser 405 nm, Argon Laser 488 nm and WL 647 nm, respectively, and detected in sequential and simultaneous acquisition settings with the HyD detectors in the gating mode. The pinhole was set to one airy unit. Image stacks were sampled with a pixel size of 142 nm and in 1 μm *z*-steps.

### Statistical analysis

Data are presented as mean ± SEM. GraphPad Prism5 was used for multiple comparisons using 2-way ANOVA with Bonferroni correction and paired t-tests. Differences were considered statistically significant at *P* < 0.05.

## Results and discussion

### Single-cell analysis revealed stage-dependent microglial clusters during neurodegeneration

**Fig. 1:**
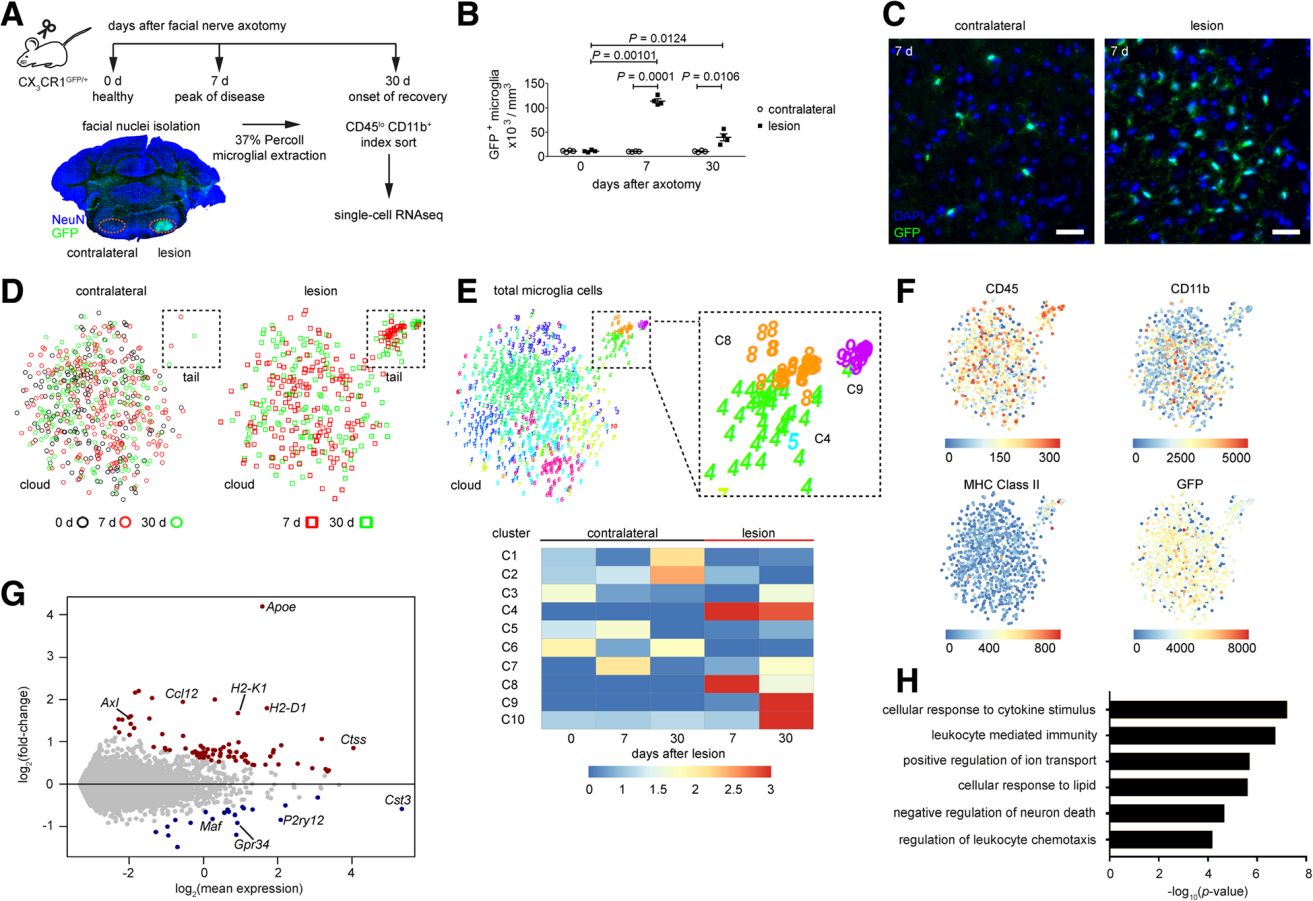
Single-cell analysis identified disease stage-specific microglial populations in a transient model of neurodegeneration. **a** Scheme of single microglial cell gene expression analysis after facial nerve axotomy (FNX) in 8 weeks old female *CX*_*3*_*CR1*^*GFP/+*^ mice. Microglia from contralateral facial nuclei (FN) of non-operated healthy mice (0 d) were used as baseline control for steady state transcriptome. Microglia from both FN of mice at peak of disease (7 d after FNX) and onset of recovery (30 d after FNX) were analyzed. A coronal brain section from 7 d after FNX at peak of disease is shown to indicate the locations of the FN (orange dotted circles) from which GFP^+^ CD45^lo^ CD11b^+^ microglia were index-sorted by FACS for RNA sequencing. **b** Quantification of GFP^+^ FN microglia after FNX. Each symbol represents mean count per animal. *N* = 4 mice per group. Two-way ANOVA and one-tailed paired t-tests showed significant difference between time and between FN at peak of disease (7 d) and onset of recovery (30 d). **c** Representative images of GFP^+^ FN microglia (green) at peak of disease (7 d) after FNX. 4′,6-diamidino-2-phenylindole (DAPI) nuclear counterstain is in blue. Scale bar: 30 μm. **d** t-distributed stochastic neighbor embedding (t-SNE) representations of 944 microglial cells from contralateral (left) and ipsilateral (right) FN based on transcriptomic analysis. The proximity of cells reflects transcriptome similarity as measured by Pearson’s correlation. Cells from contralateral FN are represented by open circles in black, red and green for disease-free (0 d), peak of disease (7 d) and onset of recovery (30 d), respectively. Cells from the injured FN are shown as open squares in red and green for 7 and 30 d and contributed significantly to the distinct “tail” population. Cells from all groups were distributed uniformly in the cloud. See Table 1 for contribution of cells per mouse. *N* = 3 mice per stage. **e** Cluster analysis based on transcriptome similarities of all 944 microglial cells revealed 10 clusters (C1-C10) of which C4 (52 cells), C8 (27 cells) and C9 (15 cells) belong to the disease-associated tail of the t-SNE map (top). Tail clusters exhibit 101 differentially expressed genes (see Additional file 1: Figure S1) in comparison to the cloud clusters (*P* < 0.05). Microglial cells from injured FN at 7 d and 30 d after FNX contribute to the tail as in (**d**). Cloud clusters (850 cells) reveal a homogeneous population of microglia in contralateral and lesion groups with minor heterogeneity in gene expression levels. The heat map shows the enrichment of microglial cells belonging to each group in clusters C1-C10 (bottom). The color legend depicts an enrichment score [−log_10_(*p*-value+ 10^− 3^)], where the *P*-value is calculated by a hypergeometric test. t-SNE representations of total FACS sorted microglia (944 cells) show the relative fluorescence intensities (color legend) of surface markers CD45, CD11b and MHC class II, and endogenous GFP mapped to single CX_3_CR1^+^ microglia. **g** MA plot of the 101 differentially expressed genes (see Additional file 1: Figure S1) that distinguish the cloud and tail (comprising C4, C8 and C9 contributed by microglial cells from injured FN at 7 d and 30 d after FNX) transcriptomes in (**e**). Ten selected upregulated (red) and down-regulated (blue) genes with a minimum of two-fold change (Benjamini-Hochberg-corrected *P* < 0.05) are indicated. Non-regulated expressed genes are shown in gray. **h** Representative Gene Ontology (GO) terms for the neurodegeneration-associated microglial gene signature represented by the 101 differentially expressed genes in (**g**)

In our previous study, we reported that the rapid increase in microglial cells in response to FNX induced neurodegeneration was due to microglial clonal expansion [43]. In addition to accompanying morphological changes in microglia of the ipsilateral FN, we identified the differential regulation of 257 microglia-expressed genes in comparison to the contralateral FN by bulk microglia RNAseq [43]. While both clonal expansion and recovery of steady state microglial cell numbers by 60 d after FNX appeared to occur randomly, it was unclear whether the molecular programming of all microglia within the lesioned FN was homogeneous. To understand the relatedness of the cells based on their transcriptomes, we performed scRNAseq of microglia isolated from the contralateral and injured FN at stages representing disease-free (0 d), peak of disease (7 d after FNX) and onset of recovery following microgliosis (30 d after FNX) (Fig. 1a-c). At least 96 microglial cells were sorted for each experimental group per animal. After quality control, data from a total of 944 cells, from which 15,245 genes were quantified, were further analyzed using our RaceID2 algorithm [16] and depicted in t-distributed stochastic neighbor embedding (t-SNE) representations (Fig. 1d-f; Table 1). Microglia from all groups distributed uniformly in the “cloud”, whereas cells that clustered separately in the “tail” were derived solely from 7 and 30 d lesion groups, indicating that the tail comprises disease stage-specific microglia (Fig. 1d). This separation of disease-associated CNS immune cell populations agrees with a recent single-cell cytometry-based study [34]. Our transcriptome-based cluster analysis of all 944 microglial cells using the RaceID2 algorithm identified ten clusters (C1-C10), of which cells from C4, C8 and C9 mapped mainly to the tail (Fig. 1e). Other clusters (C1-C3, C5-C7, and C10) were identified within the cloud representing less distinct subpopulations of microglia with variations in the expression levels of similarly expressed genes (Fig. 1e). Analysis of surface markers revealed corresponding enrichment of the activation markers CD45 and MHC class II in cells within the tail (Fig. 1f). Notably, these differences at the levels of gene and protein expression did not correlate directly to cell morphology as most microglia within the injured FN appeared to have similarly retracted their ramifications and assumed amoeboid and rod-like shapes typical of activated microglia (Fig. 1c). Differential gene expression analysis of cloud versus tail clusters (Benjamini-Hochberg-corrected *P* < 0.05) identified 101 differentially expressed genes (Fig. 1g, Additional file 1: Figure S1). Gene Set Enrichment Analysis of all genes that distinguish the cloud and tail clusters revealed that gene sets corresponding to translation, degradation of the extracellular matrix and peptide ligand-binding receptors were upregulated, whereas gene sets related to membrane trafficking, intra-Golgi and retrograde Golgi-to-ER traffic, and fatty acid metabolism were down-regulated (Additional file 2: Figure S2). The genes upregulated during neurodegeneration were enriched for the Gene Ontology (GO) terms related to immune response, lipid mediation, neuronal cell death, and migration of microglia (Fig. 1h).

**Table 1:** Contribution of facial nuclei microglia to single cell transcriptomic analysis

Group	CD45^lo^ CD11b^+^ GFP^+^ microglial cells						
	Mouse 1		Mouse 2		Mouse 3		Total
	Cloud	Tail	Cloud	Tail	Cloud	Tail	
0 d contralateral	61	2	31	0	105	4	203
7 d contralateral	76	1	77	2	60	1	217
7 d lesion	64	10	52	22	47	14	209
30 d contralateral	57	0	30	1	74	0	162
30 d lesion	37	6	30	3	50	27	153

Cloud clusters: C1-C3, C5-C7 and C10; tail clusters: C4, C8 and C9

### Common microglial gene regulatory profile across neurodegenerative diseases

**Fig. 2:**
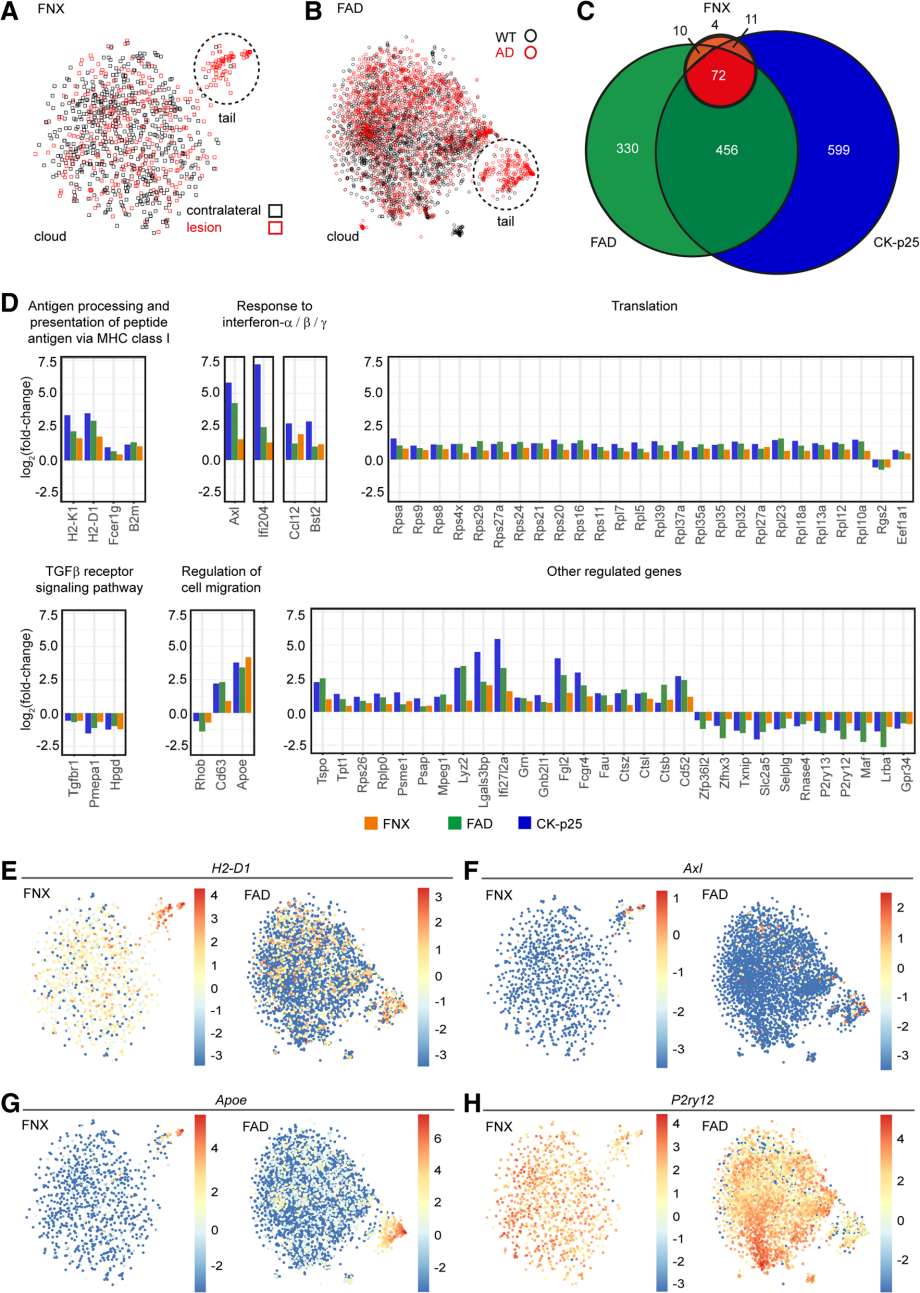
Comparative analysis of single-cell microglial transcriptomes from acute and chronic neurodegeneration models unveiled a common gene regulatory signature. **a-b** t-SNE maps of **a** 944 microglial cells from FNX acute neurodegeneration in susceptibility gene-free *CX*_*3*_*CR1*^*GFP/+*^ mice (as in Fig. 1d-e) and **b** 3896 microglial cells from chronic neurodegeneration FAD model [21] based on RaceID2 transcriptomic analysis. Cells from contralateral (black square) and lesion (red square) FN and cells from wild type (WT) controls (black circle) and AD transgenic (red circle) mice are distributed uniformly in the clouds. Neurodegeneration-associated groups formed distinct tail populations (dotted circles). **c** Comparative differential gene expression analysis of disease-associated clusters in susceptibility gene-free FNX (orange), chronic FAD (green, [21]) and severe CK-p25 (blue; [30]) neurodegeneration models identified 72 common differentially regulated genes (red). See Table 2 and Additional file 5: Table S1 for details. **d** Log_2_(fold-change) (y-axis) of 70 common neurodegeneration-associated genes (x-axis) identified in (**c**) that were similarly up- or down-regulated (represented as respective positive or negative values). Genes are categorized according to GO terms in panel titles. FNX (orange), FAD (green) and CK-p25 (blue). See also Table 2. **e-h** tSNE maps of common genes **e** *H2-D1*, **f** *Axl*, **g** *Apoe*, and **h** *P2ry12* shown in (**d**) depict their single cell expression in the FNX and FAD data sets. Color legends represent log_2_(transcript counts) across cells

The FNX paradigm represents a model for acute neurodegeneration where approximately 10–15% [7] of facial motoneurons in the FN die upon nerve transection, but clinical recovery is observed by two months [43]. We asked if our neurodegeneration-associated microglial gene signature would be identical across acute non-susceptibility gene-driven and transgene-induced chronic and destructive forms of neurodegeneration. A recent study based on scRNAseq of all immune cells in WT and AD transgenic mouse brains resulted in the identification of a unique microglia type associated with neurodegenerative diseases (DAM) [21]. The t-SNE representation of the Keren-Shaul et al. data (indicated as FAD for familial AD) resembles our FNX data set where the majority of the single-cell transcriptomes from all groups were found in the cloud while subsets of microglia from lesion or AD groups were spatially distinct in the tail (Fig. 2a-b). Here, cloud and tail transcriptomes of the AD study were distinguished by differential expression of 109 genes (Benjamini-Hochberg-corrected *P* < 0.05) of which 29 genes were common to the differentially regulated genes identified in our FNX study (Additional file 3: Figure S3). The median number of unique molecular identifiers (UMIs) detected in the FNX and FAD studies were 3660.5 and 982, respectively (Additional file 4: Figure S4). In a transgenic mouse model for severe neurodegeneration known as CK-p25, upregulation of many disease-associated genes in microglia in the late response cluster of single-cell transcriptomic analysis [30] was reminiscent of the changes we observed in our tail transcriptomes. Comparison of differentially regulated DAM genes in all three models (detailed in the Methods) revealed an overlap of 72 common genes, with only 4 genes found to be FNX-specific (Fig. 2c; Table 2 and Additional file 5: Table S1). Analysis of the fold-change of the common genes showed that 70 of the genes were correspondingly up- or downregulated (Fig. 2d-h; Table 2). Similar to the outcome of a meta-analysis of transcriptomes from aging, primed and neurodegenerative conditions [19], our finding emphasizes that despite pathology-specific contextual differences, a strong consensus neurodegeneration-associated gene signature exists (Fig. 2c; Additional file 6: Table S2). We also validated our findings against a searchable database (http://research-pub.gene.com/BrainMyeloidLandscape) that is a curated compendium of mouse and human CNS myeloid cell expression profiles from various conditions of neurodegeneration or infection [9]. Taken together, this core signature we identified may hold promising therapeutic targets for relieving severe neuronal damage in related CNS disease phenotypes.

**Table 2:**
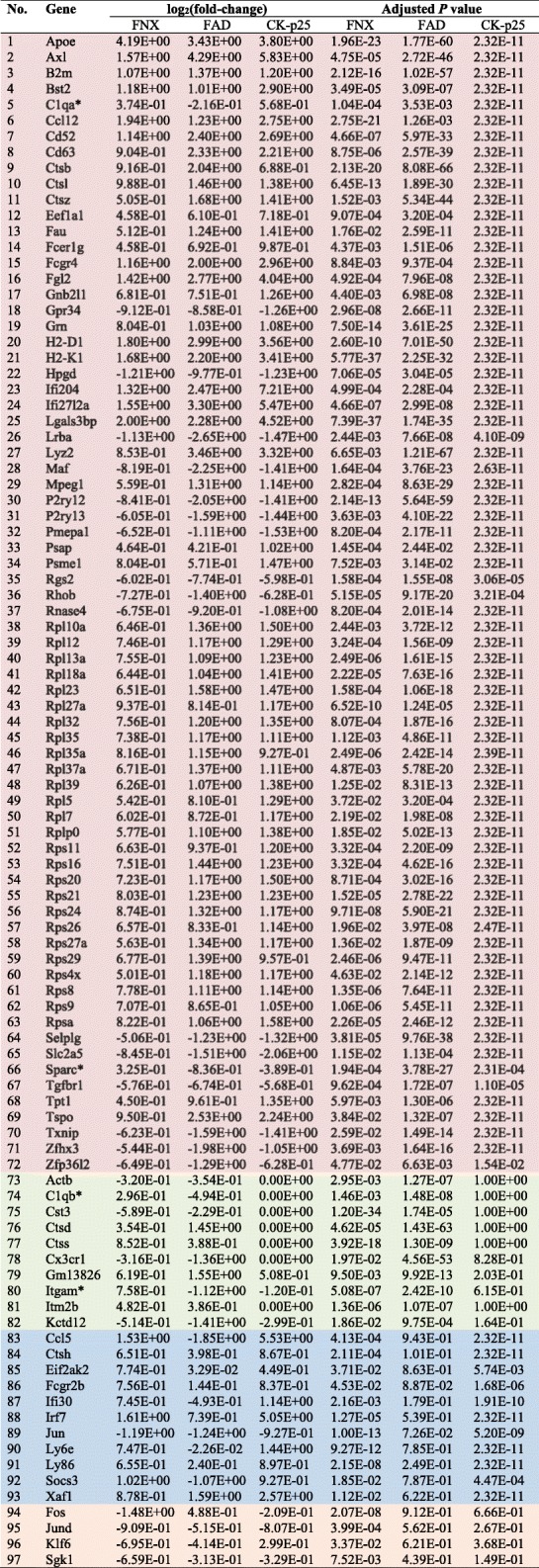
Microglia regulated disease-associated genes in models of neurodegeneration

Color code corresponds to Fig. 2c: common to FNX, FAD and CK-p25 (red); common to FNX and FAD (green); common to FNX and CK-p25 (blue); only regulated in FNX (orange). Negative values represent down-regulation. Asterisk (*) indicates different directionality of commonly regulated genes

### Recovery-associated microglial subset arises during injury resolution

**Fig. 3:**
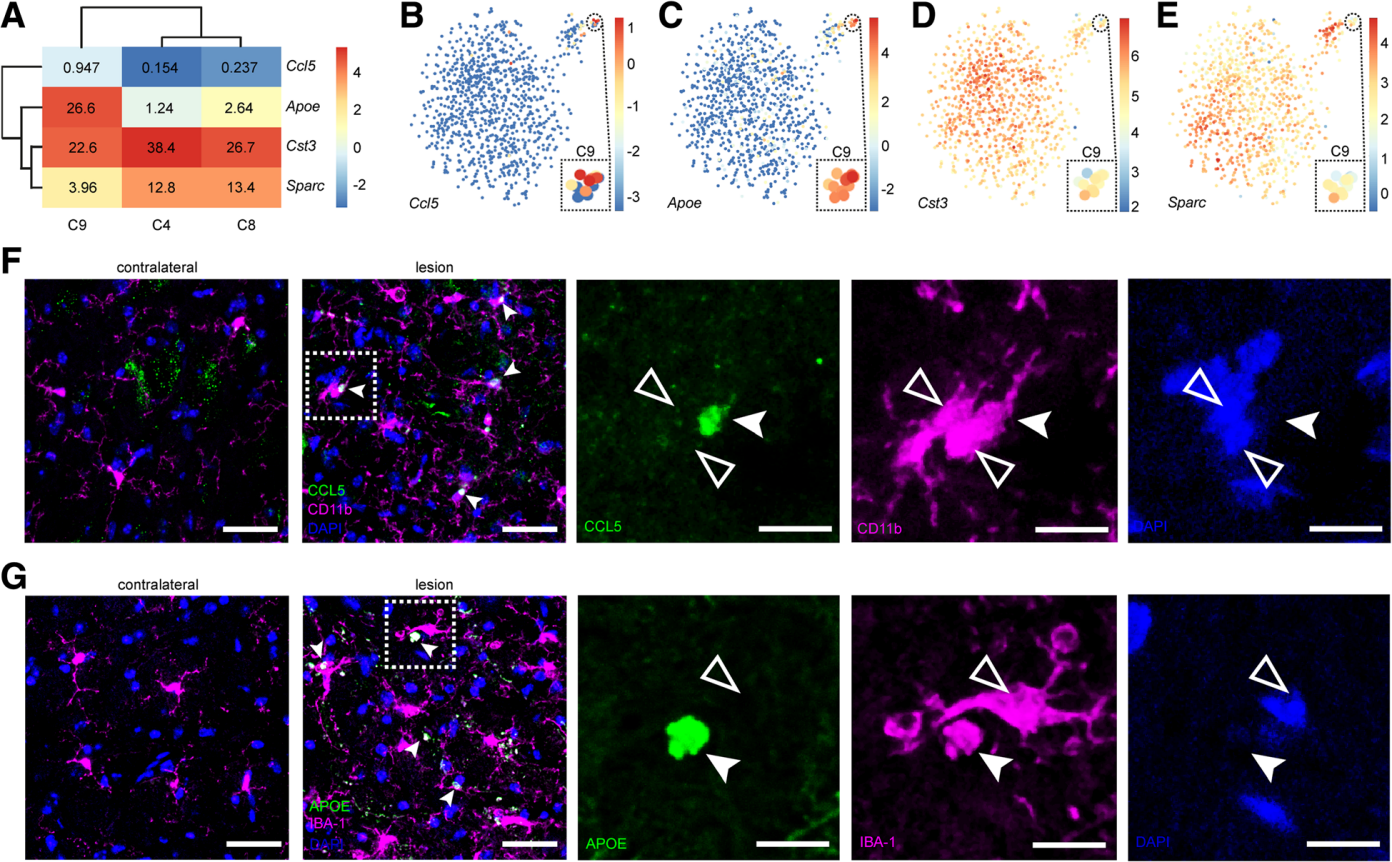
Emergence of a novel *Apoe*- and *Ccl5*-expressing microglial subset during recovery from neurodegeneration. **a** Heat map of four genes (*Ccl5*, *Apoe*, *Cst3* and *Sparc*) that distinguish the tail cluster C9 (representing onset of recovery, 30 d) from C4 and C8 (representing both peak of disease at 7 d and onset of recovery at 30 d). Mean expression values are indicated. Microglia that upregulate *Apoe* and *Ccl5* and down-regulate *Cst3* and *Sparc* map specifically to the 30 d lesion group corresponding to onset of recovery. See Fig. 1d-e. The color legend represents log_2_(transcript counts). **b-e** tSNE representations highlighting the strong upregulation of **b** *Ccl5* and **c** *Apoe* and downregulation of **d** *Cst3* and **e** *Sparc*. Transcript counts across cells are shown as log_2_(transcript counts) in color legends. **f-g** Confocal images of a subset of lesion-specific FN microglia expressing **f** CCL5 or **g** APOE (green; filled arrowhead) at the onset of recovery (30 d after FNX). Microglial cells visualized by **f** CD11b or **g** IBA-1 immunohistochemistry (magenta) that are nucleated (DAPI, blue) are indicated (open arrowhead). Scale bars, 30 μm (overview) and 10 μm (higher magnification of boxed area)

Using the FNX model, we were able to track disease progression from peak of microgliosis (7 d after FNX) to clinical recovery (60 d after FNX) that is accompanied by the resolution of microgliosis starting at 30 d [43]. Such kinetics of microgliosis are in sharp contrast to mouse models of chronic or severe neurodegeneration in which the resolution of microgliosis is not observed [21, 23, 30]. Notably, bulk RNAseq analysis revealed no change in gene regulation between the lesion and contralateral FN in at 60 d after FNX [43]. Closer examination of neurodegeneration-associated tail microglial cells revealed that cluster C9 comprises transcriptomes from the 30 d lesion group (Fig. 1d-e; Table 1). Strong upregulation of *apolipoprotein E (Apoe*) and *chemokine ligand 5* (*Ccl5*) and down-regulation of *cystatin 3 (Cst3)* and *secreted* protein *acidic and rich in cysteine or osteonectin (Sparc)* in single microglial cells distinguished C9 from C4 and C8 in the tail (Figs. 1e, 3). High expression of *Apoe* and *Ccl5* is also in agreement with our previous findings from bulk RNAseq of sorted microglia from lesioned FN at 30 d after FNX [43]. The fraction of C9 microglia to all cells from 30 d lesion (Fig. 1d; Table 1) is reflected at the level of protein expression (Fig. 3f-g). Of note CCL5^+^ CD11b^+^ and APOE^+^ IBA-1^+^ microglial cells appear amoeboid, smaller, anucleated and possibly fragmented (Fig. 3f-g), suggesting a non-homeostatic (or transient and non-propagative) phenotype.

We believe that the interpretation of microglial upregulation of APOE during brain pathology is still up for dispute. High expression of APOE has been shown to be characteristic for a subtype of reactive microglia that appears in specific conditions of neurodegeneration in mice [6, 8, 19, 21, 23]. Multiple rodent studies demonstrated that genetic deletion or repression of APOE alleviated disease severity, as observed in the amelioration of experimental autoimmune encephalomyelitis (EAE) [25, 42], extension of lifespan in the SOD1 mouse model of amyotrophic lateral sclerosis [5], and protection from tau pathogenesis typical in AD [41]. These results thus suggest a detrimental role of APOE in neurodegeneration. Studies of human brain autopsies [36] and humanized mouse models of tauopathy [41] relating to AD have however shown that different isoforms of APOE may alternate between being a risk factor or neuroprotective. Since *Apoe* is highly expressed in mouse astrocytes and microglia [47] and mainly expressed by astrocytes in human [48], it is unclear if the ablation of APOE in some or all cell types contribute similarly to CNS pathology. In agreement with the observation that the upregulation of *Apoe* during the initial DAM activation in the FAD model is independent of triggering receptor expressed on myeloid cells 2 (Trem2) [21], the FNX-dependent upregulation of *Apoe* in cluster C9 from the onset of recovery corresponds with no change in *Trem2* expression (Additional file 5: Table S1). Our immunohistochemical results depicting C9 microglia that upregulate APOE (Fig. 3g) during recovery support the claim that switching on the TREM2-APOE pathway drives a non-homeostatic microglial phenotype [23]. However, could the higher frequency of *Apoe* upregulation during early disease stage in the FAD model [21] represent a recovery-promoting microglial subtype? In the EAE study, overall levels of APOE transcript and protein in rat spinal cord reduced at onset, elevated during peak, and plateaued at the end of disease [25]. Notably, a brain region-specific proteomic investigation of APOE protein levels and amyloid accumulation in three AD mouse models led the authors to predict that increased APOE detection drove amyloid clearance [40]. There are few clues to date regarding the functional or mechanistic role of the chemoattractant and activating cytokine CCL5 or RANTES [17] particularly in microglia. The down-regulation of *Cst3* seems to imply a loss of homeostatic microglial phenotype since it is typically considered a microglia signature gene [21]. Astrocyte-secreted SPARC protein was described to be antagonistic to synaptogenic function [24], however it is presently unclear if the down-regulation of *Sparc* in microglia during recovery plays a supportive role for synaptogenesis. Overall, it remains to be investigated whether microglia carrying the recovery-associated gene signature are targeted for removal by local apoptosis and/or emigration [43] during reinstatement of steady state microglial network that accompany CNS regeneration.

## Conclusion

In conclusion, our combinatorial analysis of microglia gene expression profiles across neurodegeneration models strongly implicates APOE in disease modulation. However, our FNX model opens a new window for further investigation into the significance of this and other pathways during microglia-directed disease amelioration and recovery of CNS health.

## Additional files

Additional file 1: **Figure S1.** Heat map of 101 differentially expressed genes that distinguish the cloud and tail (comprising C4, C8 and C9) transcriptomes from the FNX neurodegeneration model depicted across clusters 1 to 10 based on Benjamini-Hochberg-corrected *P* < 0.05. The color legend represents log_2_(transcript counts). (TIF 25531 kb)

Additional file 2: **Figure S2.** Gene Set Enrichment Analysis of all differentially expressed genes that distinguish the cloud and tail (comprising C4, C8 and C9) transcriptomes from the FNX neurodegeneration model depicted across clusters 1 to 10 based on Benjamini-Hochberg-corrected *P* < 0.1. The color legend represents the *P*-values. (TIF 25527 kb)

Additional file 3: **Figure S3.** MA plot of the 101 differentially expressed genes that distinguish the cloud and tail (in Fig. 1d-e) in the FNX neurodegeneration model. Genes that also distinguish the cloud and tail clusters in the FAD data set (in Fig. 2b) are indicated here as common genes (blue). Upregulated (green) and down-regulated (red) genes with a minimum of two-fold change (Benjamini-Hochberg-corrected *P* < 0.05) and non-regulated expressed genes (gray) are shown. (TIF 25538 kb)

Additional file 4: **Figure S4.** Box plot of unique molecular identifiers (UMIs) detected from each single microglial cell in the FNX and FAD neurodegeneration models. (TIF 25520 kb)

Additional file 5: **Table S1.** Fold changes and adjusted *P* values of all 5820 genes included in the comparative transcriptomic analysis. (XLSX 479 kb)

Additional file 6: **Table S2.** Overlap of the 72 common differentially regulated genes (Fig. 2 and Table 2) with the consensus microglia gene expression signature induced by aging, primed and neurodegenerative conditions reported in Table S5 [19]. (XLSX 13 kb)
