# Supplementary figures and images for: Unique microglia recovery population revealed by single-cell RNAseq following neurodegeneration
Source: Acta Neuropathol Commun. 2018 Sep 5;6:87. doi: 10.1186/s40478-018-0584-3 (PMC6123921; doi:10.1186/s40478-018-0584-3)

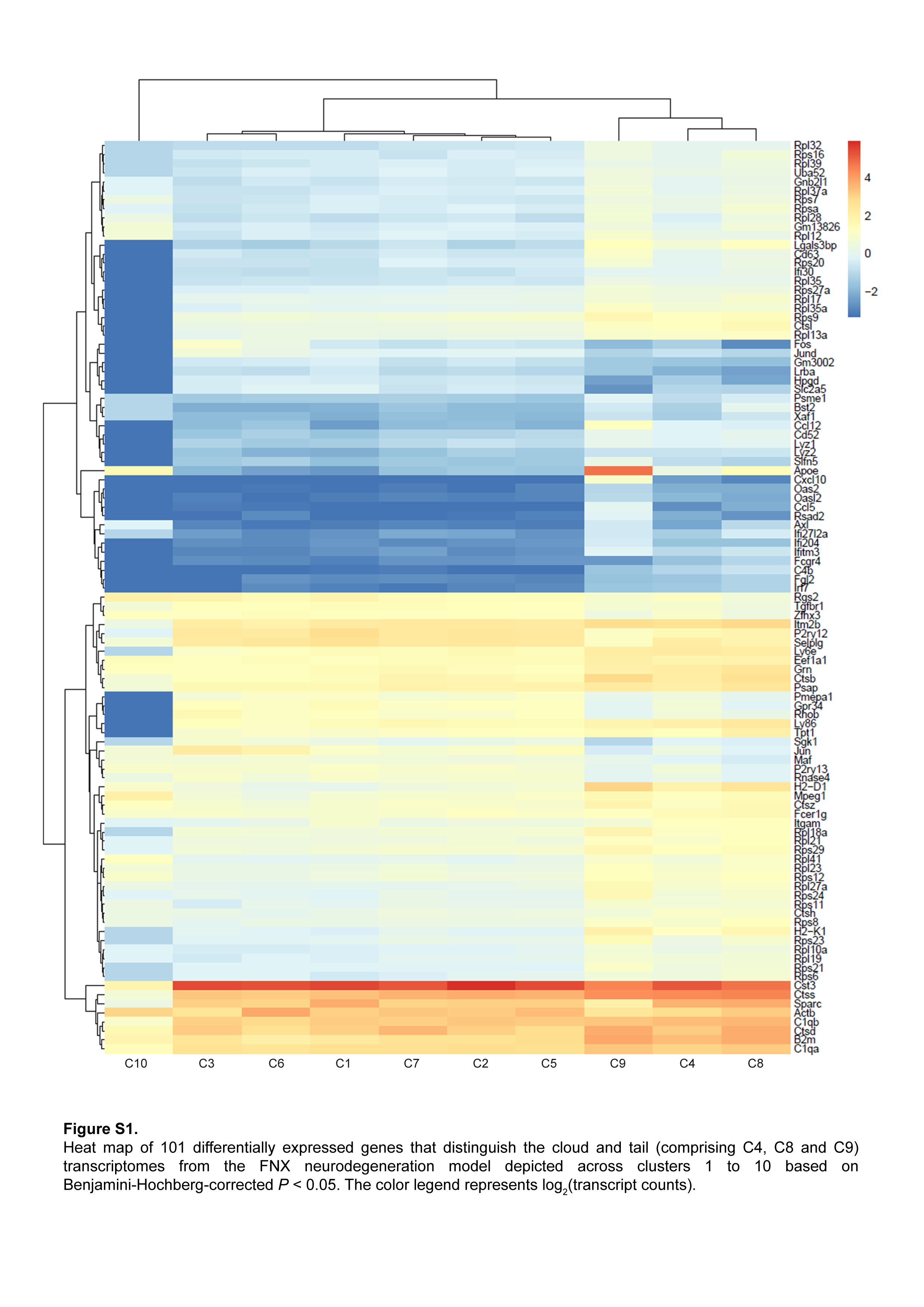

Supplement: Supplementary file 1 — Figure S1. Heat map of 101 differentially expressed genes that distinguish the cloud and tail (comprising C4, C8 and C9) transcriptomes from the FNX neurodegeneration model depicted across clusters 1 to 10 based on Benjamini-Hochberg-corrected P < 0.05. The color legend represents log2(transcript counts). (TIF 25531 kb) [file 40478_2018_584_MOESM1_ESM.tif]

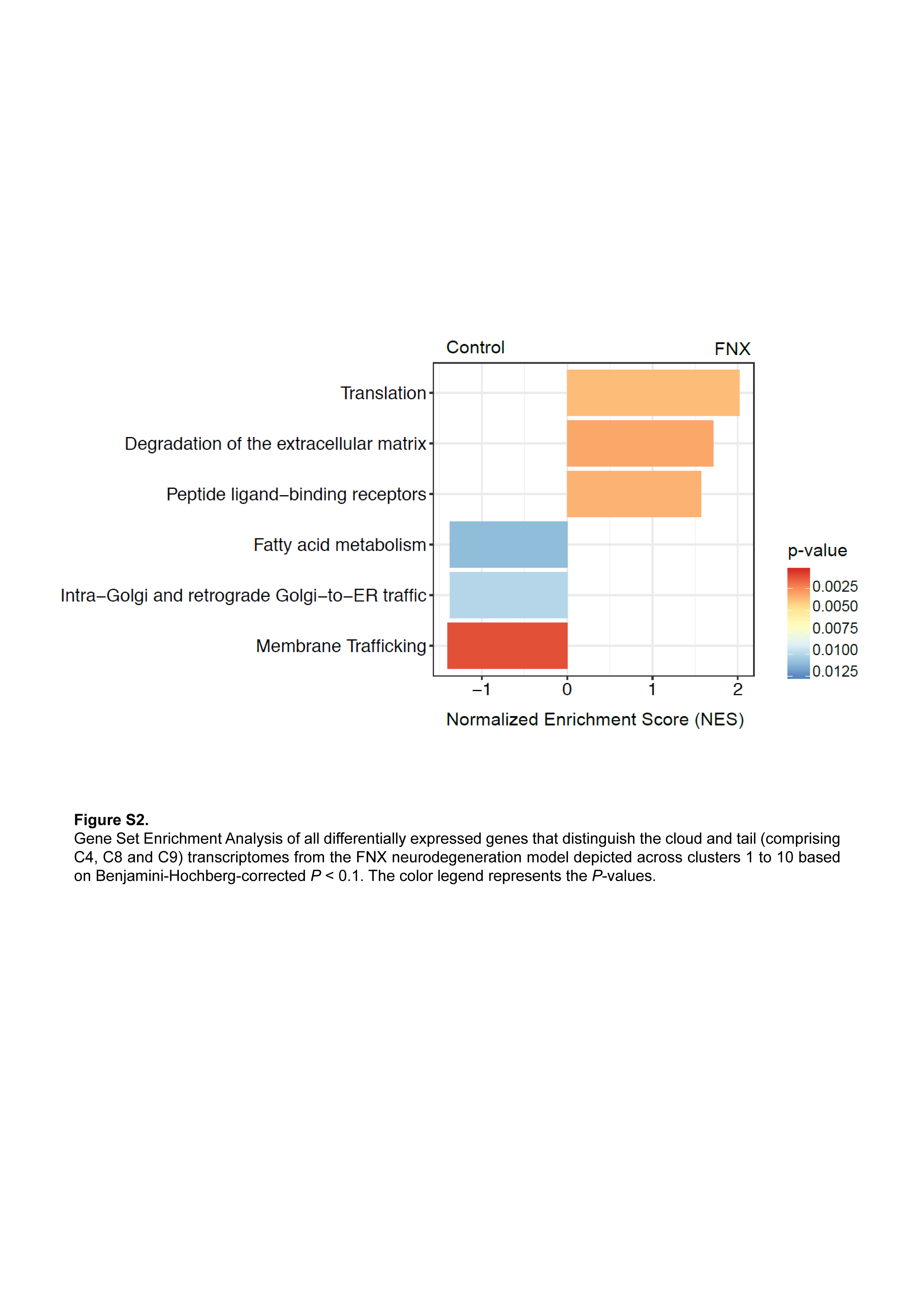

Supplement: Supplementary file 2 — Figure S2. Gene Set Enrichment Analysis of all differentially expressed genes that distinguish the cloud and tail (comprising C4, C8 and C9) transcriptomes from the FNX neurodegeneration model depicted across clusters 1 to 10 based on Benjamini-Hochberg-corrected P < 0.1. The color legend represents the P-values. (TIF 25527 kb) [file 40478_2018_584_MOESM2_ESM.tif]

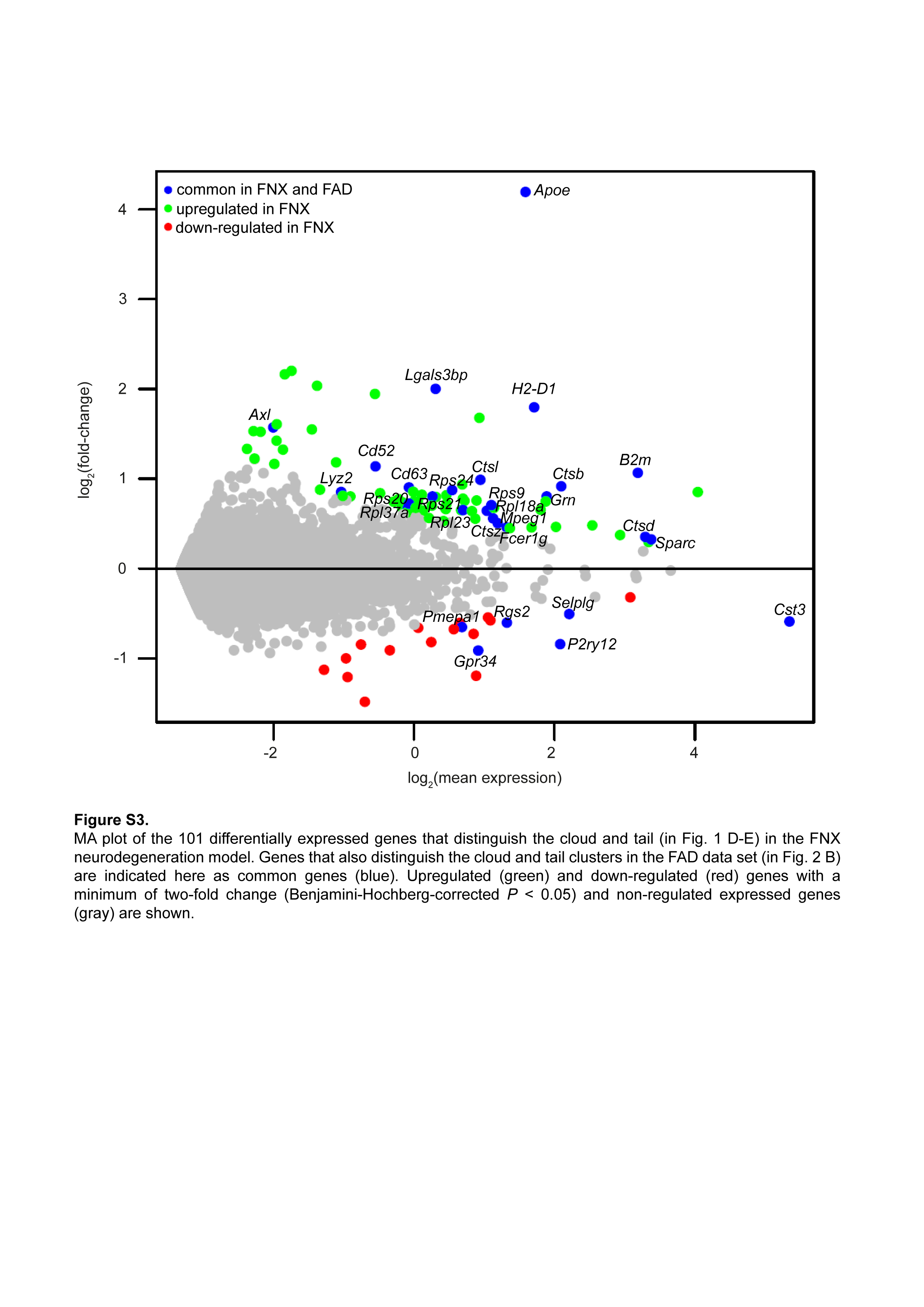

Supplement: Supplementary file 3 — Figure S3. MA plot of the 101 differentially expressed genes that distinguish the cloud and tail (in Fig. 1d-e) in the FNX neurodegeneration model. Genes that also distinguish the cloud and tail clusters in the FAD data set (in Fig. 2b) are indicated here as common genes (blue). Upregulated (green) and down-regulated (red) genes with a minimum of two-fold change (Benjamini-Hochberg-corrected P < 0.05) and non-regulated expressed genes (gray) are shown. (TIF 25538 kb) [file 40478_2018_584_MOESM3_ESM.tif]

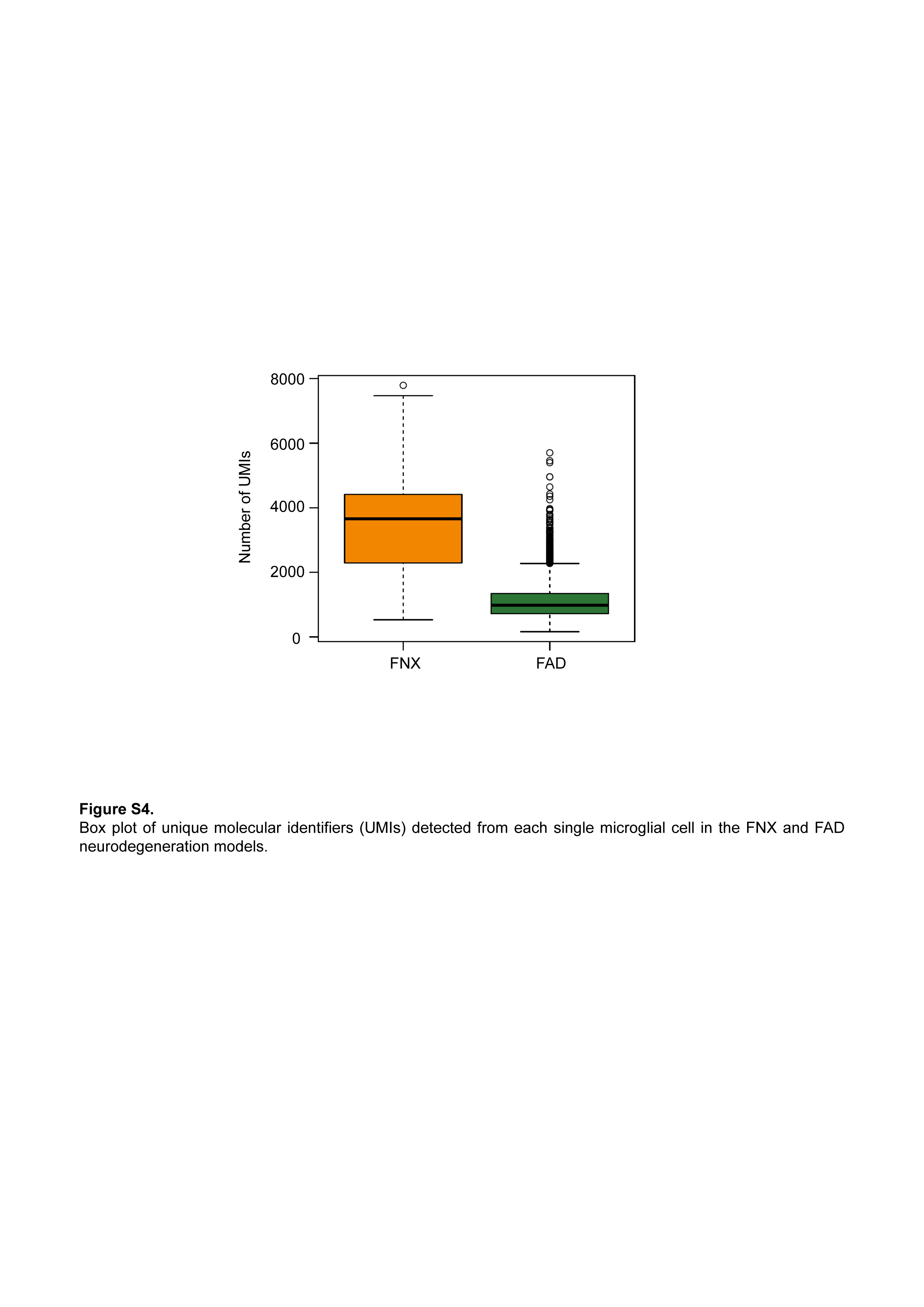

Supplement: Supplementary file 4 — Figure S4. Box plot of unique molecular identifiers (UMIs) detected from each single microglial cell in the FNX and FAD neurodegeneration models. (TIF 25520 kb) [file 40478_2018_584_MOESM4_ESM.tif]
